# Polypropylene microplastics promote metastatic features in human breast cancer

**DOI:** 10.1038/s41598-023-33393-8

**Published:** 2023-04-17

**Authors:** Jun Hyung Park, Seungwoo Hong, Ok-Hyeon Kim, Chul-Hong Kim, Jinho Kim, Jung-Woong Kim, Sungguan Hong, Hyun Jung Lee

**Affiliations:** 1 Department of Global Innovative Drugs, Graduate School of Chung-Ang University, Seoul, 06974 South Korea; 2 Department of Chemistry, College of Natural Sciences, Chung-Ang University, Seoul, 06974 South Korea; 3 Department of Anatomy and Cell Biology, College of Medicine, Chung-Ang University, Seoul, 06974 South Korea; 4 Department of Life Sciences, Chung-Ang University, Seoul, 06974 South Korea

**Keywords:** Biochemistry, Cancer, Environmental sciences, Oncology, Materials science

## Abstract

Microplastics (MPs) are now a global issue due to increased plastic production and use. Recently, various studies have been performed in response to the human health risk assessment. However, these studies have focused on spherical MPs, which have smooth edges and a spherical shape and account for less than 1% of MPs in nature. Unfortunately, studies on fragment-type MPs are very limited and remain in the initial stages. In this study, we studied the effect that 16.4 µm fragment type polypropylene (PP) MPs, which have an irregular shape and sharp edges and form naturally in the environment, had on breast cancer. The detrimental effects of PPMPs on breast cancer metastasis were examined. Here, 1.6 mg/ml of PPMP, which does not induce cytotoxicity in MDA-MB-231, was used, and at this concentration, PPMP did not induce morphological changes or cellular migrating in the MDA-MB-231 and MCF-7 cells. However, PPMP incubation for 24 hours in the MDA-MB-231 cells significantly altered the level of cell cycle-related transcripts in an RNA-seq analysis. When confirmed by qRT-PCR, the gene expression of TMBIM6, AP2M1, and PTP4A2 was increased, while the transcript level of FTH1 was decreased. Further, secretion of the pro-inflammatory cytokine IL-6 from cancer cells was elevated with the incubation of PPMP for 12 hours. These results suggest that PPMP enhances metastasis-related gene expression and cytokines in breast cancer cells, exacerbating breast cancer metastasis.

## Introduction

Microplastics (MPs) are typically less than 5 mm in size and are not only derived from the degradation of plastic objects in the environment but are also produced for commercial uses^1,2^. Recently, MPs have been deemed a new environmental pollutant due to their increased production and extremely low natural biodegradation in the ecosystem^3^. There are increasing reports of MPs in drinking water and food products, including sea salt, due to their broad range in shape and size and the number of polymers in the oceans and freshwater ecosystems^4–6^. Importantly, MPs can enter the human body via the accumulation of MPs in the food chain. It was reported that MPs had been detected in the gastrointestinal tract of marine animals and human tissue and organs^7,8^.

MPs pose significant environmental and health concerns because of their persistence in the environment, potential toxicity, and ability to absorb contaminants and pathogens from the environment. However, the long-term effects of MPs on human health are currently unknown^9^. Most studies that examined the health effects of MPs have used animal subjects. The ingestion of MPs has been shown to cause an inflammatory response and can damage the gut, disrupt gut microorganisms, cause organ damage, and affect reproduction and metabolism. Breathing in MPs can cause inflammation and chemical toxicity and introduce pathogenic microorganisms into the body. MPs introduced through skin contact can cause skin damage due to local inflammation and cellular toxicity^10^.

Notably, the use of face masks made of synthetic fibers and disposable food containers during the coronavirus disease of 2019 (COVID-19) pandemic may have contributed to the exposure of MPs^11,12^. Further, most plastic products have been known to release estrogenic chemicals or endocrine-disrupting chemicals (EDCs)^13^. Exposure to EDCs and estrogenic chemicals may increase the risk of cancer or metabolic syndromes, including obesity^11,14^. Currently, there is a lack of evidence on the actual risks of MPs relating to cancer development or metabolic diseases, despite claims in scientific articles. This study evaluated the effect of polypropylene MPS (PPMPs) on human breast cancer cells. We found that moderate amounts of PPMPs significantly accelerated the cell cycle of cancer cells and enhanced the secretion of interleukin 6 (IL-6) in the human breast cancer cell lines, MDA-MB-231 and MCF-7; however, cellular migration and motility were not altered. In addition, our RNAseq analysis revealed that PPMPs affected cancer cell-matrix adhesion and cell cycle-related signaling in human breast cancer. Consequently, chronic exposure to PPMPs may increase the risk of cancer progression and metastasis.

## Methods

### Preparation of PPMPs

Polypropylene pellets (PPs) were purchased from the Namyang Corporation (Korea). Pellets were crushed thoroughly by a cryogenic grinder. We used a 6875D Freezer/Mill (SPEX SamplePrep, United States) at the National Instrumentation Center for Environmental Management, Seoul National University (Korea). Then, the powder from the crushed PPs was filtered and collected based on particle size using a 100 μm-micropore nickel base sieve (Precision Eforming, United States).

### Particle size analysis

The particle size distribution plot of the PPs used in this study was calculated based on the data from the particle size analyzer. We used a Mastersizer 3000 (Malvern Panalytical, United Kingdom) at the Center for Biotechnology Facilities, Chung-Ang University (Korea). The automatic dry dispersion method was selected to detect the most pristine form of the MP particles.

### Scanning electron microscopy (SEM)

The medium was replaced with medium with or without PPMPs at 1.6 mg/ml, and incubated at 37 °C in 5% CO_2_ for 24 hours. Cells were washed with phosphate buffered saline (PBS) and fixed in 2% glutaraldehyde (Sigma-Aldrich, USA) for 1 hour. The cells underwent a graded ethanol dehydration series (50, 75, 90, 95, 100%) for ten minutes each. Cells were subsequently immersed in a 1:1 ethanol/hexamethyldisilazane (HMDS, Sigma-Aldrich, USA), 1:2 ethanol/HMDS, and 100% HMDS for 5 minutes each. The samples were mounted on stubs, coated with a layer of platinum, and viewed using an S-3400N microscope (Hitachi, Japan) at 10 kV.

### High-performance liquid chromatography–diode array detection (HPLC–DAD) analysis

All samples were analyzed using an Agilent 1260 infinity II high-performance liquid chromatography/diode array detector (HPLC/DAD) system consisting of quaternary pumps (G7111A), a G7129A autosampler, a G7116A column oven, and a G7115A diode array detector. Data analysis was performed by the Agilent openlab CDS chemstation software (version: C.01.10[201]). HPLC analysis was performed on an Eclipse XDB-C18 column (250 mm × 4.6 mm, 5 μm). Isocratic elution was carried out using a mobile phase consisting of a mixture of 0.1% acetic acid in water and 0.1% acetic acid in acetonitrile (a ratio of 70:30). The monitoring wavelength ranged from 200 to 400 nm. Detection was performed using wavelengths of 210, 254, and 350 nm. An injection volume of 10 μL was used. The total running time was 60 minutes.

### Cell culture

The human breast cancer cell lines MCF-7 and MDA-MB-231 were purchased from the American Type Culture Collection (ATCC, USA). MCF-7 cells were cultured in Dulbecco’s modified essential medium (DMEM, Welgene, Korea) supplemented with 10% fetal bovine serum (FBS, Abfrontier, Korea) and 1% penicillin/streptomycin (Gibco, USA). MDA-MB-231 cells were cultured in Roswell Park Memorial Institute medium (RPMI) 1640 (Welgene, Korea) containing 10% FBS and 1% penicillin/streptomycin at 37 °C in 5% CO_2_ (v/v). Sterilization was performed in consideration of the melting point of polypropylene (about 160 degrees). Before the experiment, PPMPs were sterilized by autoclaving at 121 °C for 15 minutes and UV treatment for 30 minutes. Sterilized PPMPs were mixed with cell medium at a concentration of 1.6 mg/ml and sonicated for 30 minutes to be added to the cell culture. The observed PPMPs did not aggregate together.

### Cell viability

Cell viability was assessed using the AlamarBlue assay kit (Invitrogen, USA) following the manufacturer’s instructions. MDA-MB-231 cells were seeded onto 96-well culture plates (Thermo Fisher Scientific, USA) at a density of 5 × 10^3^ cells per well and allowed to adhere overnight. Various concentrations (0, 0.4, 0.8, 1.2, 1.6 and 2.0 mg/ml) of PPMPs were added to each well, and the cells were incubated for 24 hours. At the end of the experiment, 10% (v/v) AlamarBlue solution was added to each well. After a 2-hour incubation, the fluorescence intensity was measured by a Synergy LX Multi-Mode Reader (Agilent Technologies, USA) at excitation and emission wavelengths of 530 and 590 nm, respectively. All assays were performed with three replicates.

### Time-lapse imaging

For the cell migration tracking experiments, a six-well plate (SPL, Korea) was placed on an inverted microscope (Eclipse Ti2, Nikon, Japan). The medium was replaced with medium with or without PPMPs at 1.6 mg/ml, and then breast cancer cells, MDA-MB-231 and MCF-7 motility was observed. Images were recorded every three minutes for 6 hours in a chamber maintained at 37 °C with 5% CO_2_. Cells were tracked by time-lapse image sequences using the manual tracking plug-in for Image J (http://rsb.info.nih.gov/ij). The Image J output was integrated into the Chemotaxis and Migration Tool software (ibidi GmbH, Germany) to determine the migration position and velocity of the cells in each well. The total distances and average velocities were obtained from 360 cells for each group (30 cells for each position, 4 positions per sample, 3 independent experiments).

### Wound healing

For the wound healing migration assay, cells were seeded onto a 24-well plate and allowed to grow to form a confluent monolayer. MDA-MB-213 and MCF-7 human breast cancer cells were wounded with a 200 µL pipette tip. After wounding, the detached cells were removed, the medium was replaced with culture media containing a vehicle or 1.6 mg/ml PPMPs, and cells were further incubated for 12 hours. Images of the wound gap were monitored under a phase-contrast microscope (IX-81, Olympus, Japan), and the wound closure was measured using Image J software (http://rsb.info.nih.gov/ij).

### RNA-sequencing and bioinformatic analysis

The RNA-seq library was generated using the TruSeq mRNA Library Prep Kit (Illumina, Inc., USA) according to the manufacturer’s instructions. Briefly, total RNA (50 ng) from the MDA-MB-231 cells (which treated MP medium for 24 hours the previous day) was isolated, a 5’ oligo-dT primer was hybridized to the RNA, and reverse transcription was carried out. Second-strand synthesis was initiated with a random primer containing an Illumina-compatible linker sequence at its 5’ end. The double-stranded library was purified with AMPure magnetic beads (A63881, Beckman coulter, CA, USA) to remove all the reaction components. High-throughput sequencing was carried out as paired-end 101 sequencing reads using a NovaSeq 6000 (Illumina, Inc., USA). mRNA-Seq reads were aligned using STAR-2.7.1a. The alignment file was applied for assembling transcripts, estimating their abundances, and detecting the differential expression of genes. We perform read feature counting with RNA-Seq by expectation-maximization (RSEM) (v.1.3.3), and the fragments per kilobase of transcript per million fragments mapped (FPKM) were calculated using the FPKM function from DESeq2 (v.1.32.0). The differential transcripts and genes expression were filtered by absolute log2 fold-change [log2 FC] > 1 and p-value < 0.01 by edgeR (v.3.36.0). The significant DEGs/DETs were found by more strict criteria abs(log2 FC > 1.5) and p-value < 0.01. Gene ontology (GO) and pathway analysis were performed by DAVID (PMID: 35325185).

### qRT-PCR

Total RNA was extracted from 8 × 10^5^ of MDA-MB-231 and MCF-7 cells using the RNeasy Mini kit (QIAGEN, Germany), according to the manufacturer’s instructions. Reverse transcription of the RNA was performed using the Maxima First Strand cDNA Synthesis Kit for qRT-PCR (Thermo Fisher Scientific, USA). Quantitative real-time polymerase chain reaction (qRT-PCR) was performed using the Power SYBR Green PCR Master Mix reagent (Applied Biosystems) and StepOne Plus real-time PCR kit (Applied Biosystems). For calculating the fold change, the cycle thresholds (Cts) were determined using the StepOne software v.2.3 (Applied Biosystems), and mRNA expression was normalized to the GAPDH transcript and the control sample. The primer sequences of the various genes were as follows: TMBIM6 (Forward: 5′- AGAGCTTCAGTGTGAGAGGA-3′; Reverse: 5′- GCAGCTAAATCAGAGGACAG-3′), AP2M1 (Forward: 5′- CAAGCAAGAGCGGGAAGC-3′; Reverse: 5′- CCATCTGGCGGGATAAAGC-3′), PTP4A2 (Forward: 5 GGAATCCACGTTCTAGATTGGC-3′; Reverse: 5′- AACACAGCAACCTGGCTCTT-3′), FTH1 (Forward: 5′- CTCCTACGTTTACCTGTCCA-3′; Reverse: 5′- CTCTCCCAGTCATCACAGTC-3′), and GAPDH (Forward: 5′-CAGCCTCAAGATCATCAGCA-3′; Reverse: 5′-TGTGGTCATGAGTCCTTCCA-3′).

### Enzyme-linked immunoassay (ELISA)

Cells were seeded in 100 mm culture dishes at a density of 8 × 10^5^ cells/ml, cultured until 90% confluence, and then replaced with or without PPMPs medium at a concentration of 1.6mg/ml. Following a 24-hour incubation at 37 °C, the supernatant was collected and analyzed for IL-6 levels using the CymaxTM Human IL-6 enzyme-linked immunoassay (ELISA) KIT (Abfrontier, Korea) following the manufacturer’s instructions.

### Cell-cycle progression with immunofluorescence/proliferation

The cell cycle analysis was performed using a Premo FUCCI Cell Cycle Sensor BacMam 2.0 (Thermo Fisher Scientific, USA). MCF-7 cells were seeded on a six-well plate at a density of 10^4^ cells per well. When the cells were completely attached, the Premo™ reagent (geminin-GFP, Cdt1-RFP), at a volume of 20 particles per cell, was transduced into the cells. After 16 hours of processing the Premo™ reagent, the media was replaced with a medium with or without 1.6mg/ml PPMPs, and consecutive images were recorded every 5 minutes for 24 hours in a chamber maintained at 37 °C with 5% CO_2_.

### Statistical analysis

Statistical analyses were performed using GraphPad Prism Version 9 (GraphPad, Inc., USA). All data were reported as the mean ± standard error of the mean (*SEM*), and *p*-values were determined using an unpaired *t* test or one-way analysis of variance (ANOVA). Significant differences between the groups were established at the following *p-*values: * *p* < 0.05, ** *p* < 0.01, *** *p* < 0.001, and **** *p* < 0.0001.

## Results

### Characterization of polypropylene microplastics (PPMPs)

**Figure 1:**
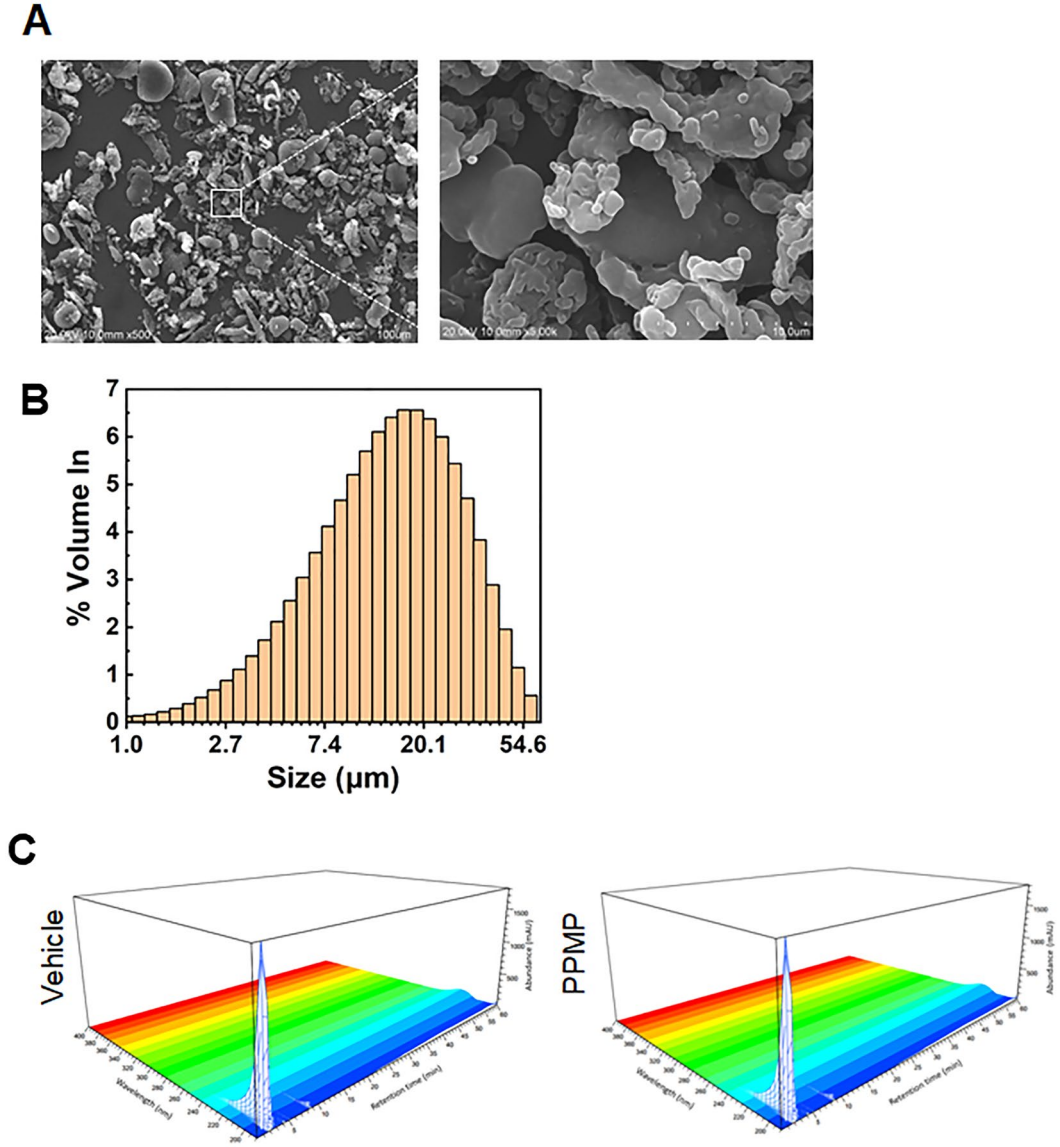
Characterization and SEM images of the PPMPs (**A**) SEM images of the fragment type PPMPs (**B**) The particle size distribution plot of the PPMPs indicates that the PP particles have the largest volume density (%) at 16.40 μm. (**C**) Comparison of the HPLC chromatograms before (left, Vehicle) and after the microplastic treatment at the selective adsorptions (right, PPMP).

The SEM images were used to confirm the morphology of the PPMP fragment types, and a variety of shapes were observed (Fig. 1A). The most frequent size of the PPMP fragments was around 16.4 µm (Fig. 1B). We did not separate out the larger-sized PPMPs (more than 20 µm) because MPs produced in the real world exist in various sizes and shapes, and humans do not use different pathways to uptake the different sized and shaped PPMPs but indiscriminately uptake PPMPs smaller than 200 µm in size. Therefore, we focused on studying MPs similar to what exists in the real world.

Generally, plastic products contain artificial synthetic chemicals, such as processing aids, plasticizers, reinforcing agents, light stabilizers, oxidizing agents, flame retardants, and foaming agents. Therefore, we examined the contribution of the chemicals leaked from the MPs used in this study. To evaluate these chemicals, we added fragmented MPs to the complete medium and incubated them for 72 hours. We then detected the extracted chemicals from the MPs using HPLC/DAD. There was no significant difference between the extracted chemicals from the vehicle and MPs (Fig. 1C). These results suggest that chemicals were not extracted from the MPs during the incubation. Therefore, all the physiological observations in this study were effects of the MPs.

### PPMPs did not show cytotoxicity

**Figure 2:**
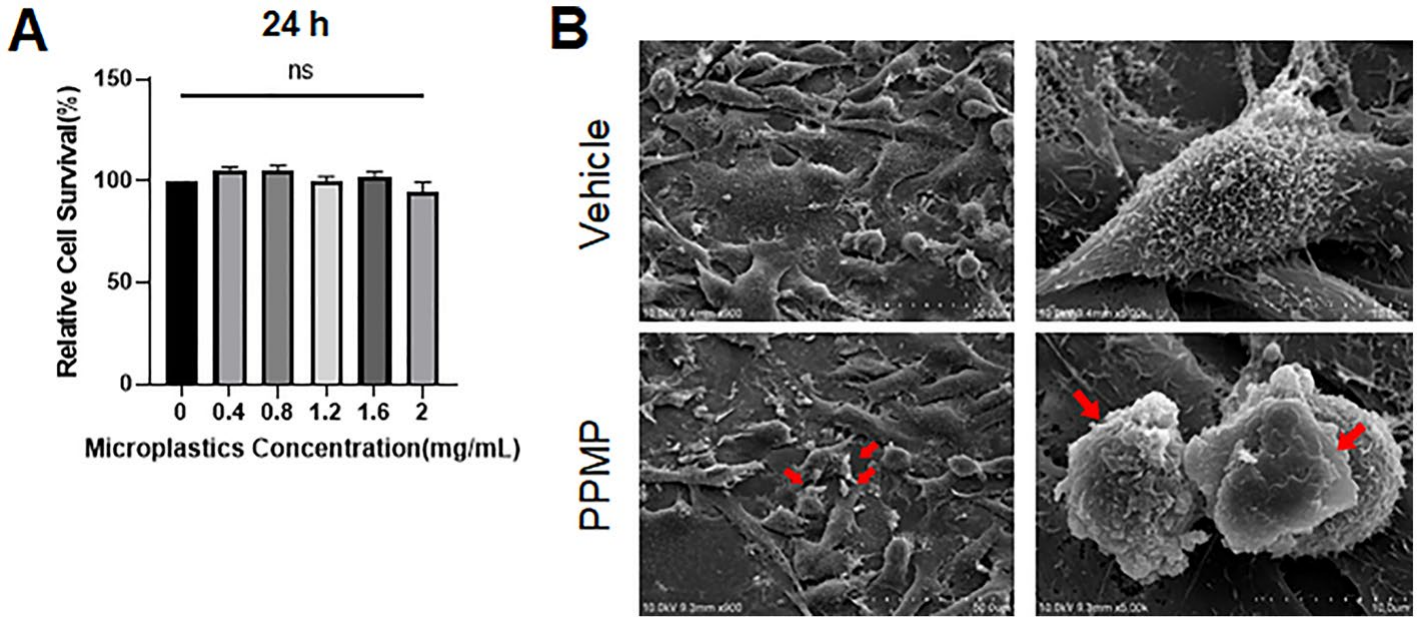
Effect of PPMP on cell viability in human breast cancer cells. (**A**) PPMPs were added to the MDA-MB-231 cells and incubated for 24 hours. The graph shows the survival rates of cells with or without PPMPs (n = 3 independent experiments; one-way ANOVA, *p < 0.05, **p < 0.01 relative to 0 mg/ml PPMPs). (**B**) SEM image shows MDA-MB-231 cells with or without PPMPs for 24 hours. The red arrows indicate a PPMP on the cell surface.

In order to assess the cytotoxicity of the MPs, PPMPs were added to MDA-MB-231 cells at various concentrations (0, 0.4, 0.8, 1.2, 1.6, and 2 mg/ml). The cell viability was measured 24 hours after the administration of the PPMPs. The cell viability was not affected by the PPMP treatment regardless of their concentration, suggesting that the PPMPs did not induce cytotoxicity in these breast cancer cells (Fig. 2A). For subsequent experiments in this study, we wanted to apply the highest concentration of PPMPs that did not affect the viability of the MDA-MB-231 cells. Although the PPMP-induced toxicity was not statistically significant, the cell viability was slightly decreased at 2.0 mg/ml (94.5%) compared with 1.6 mg/ml (102.4%), so all subsequent experiments were performed using 1.6 mg/ml PPMPs. After adding 1.6 mg/ml PPMPs for 24 hours, the cells were fixed and subjected to SEM imaging. PPMPs were sitting down to the surface of cells or inserted between cells (Fig. 2B). Altered cellular shape and morphology were not observed in the ultrastructural analysis. Since most of the PPMPs tended to float in the medium when treated, small portion of PPMPs were sunken to the cellular membrane or the bottom of the plate (Fig. 2B).

### PPMPs did not alter cellular motility in breast cancer cells

**Figure 3:**
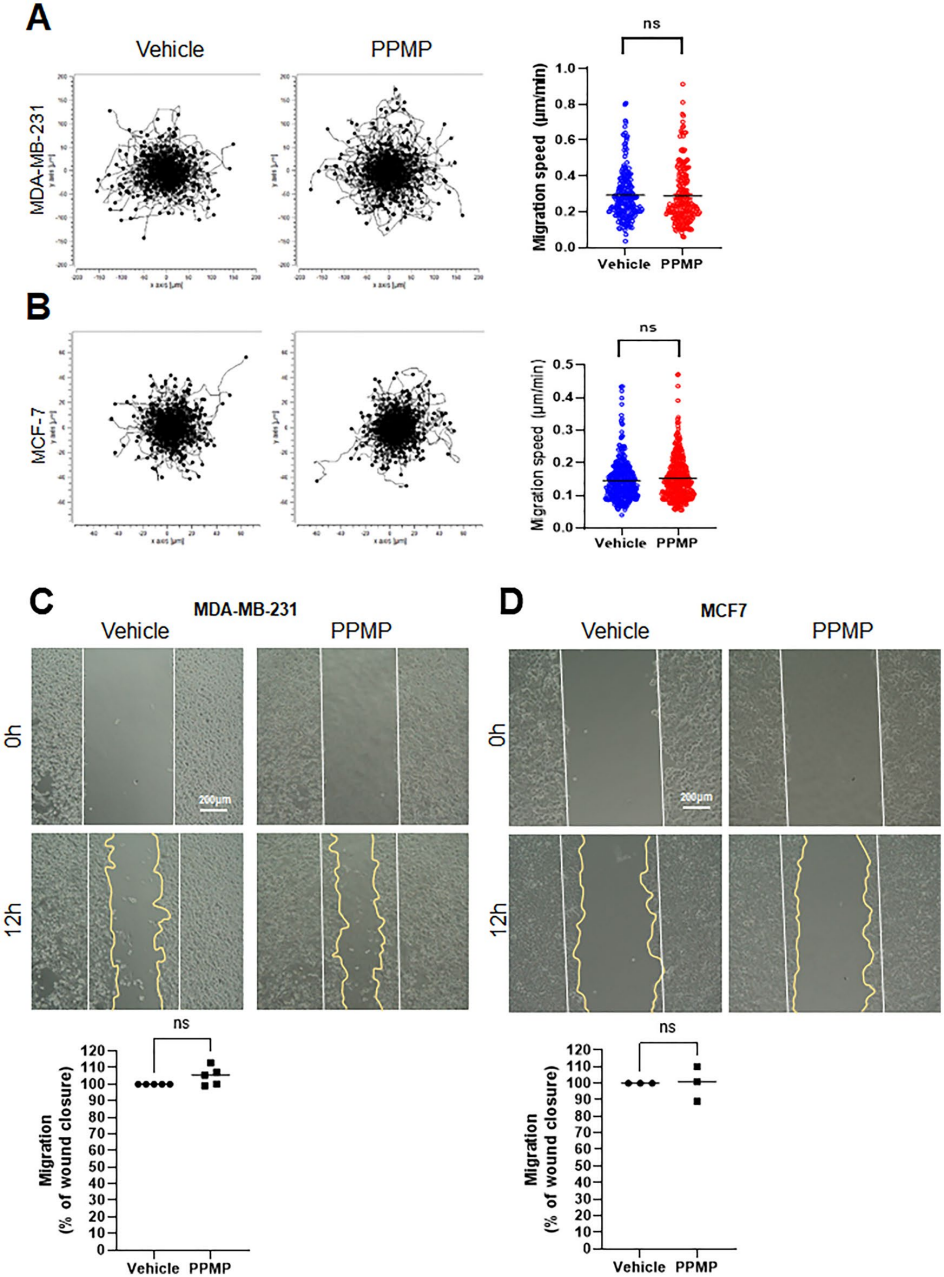
Effect of PPMP on breast cancer cell motility. (**A**) Both breast cancer cell lines were incubated with PPMPs and observed under a time-lapse imaging microscope for six hours. The plots depict the motility of individual cells in the representative experiment. (**B**) Quantification of the migration speed reveals unchanged cellular velocity by the PPMPs treatment in both cell lines. The results are expression as the mean ± *SEM*; n = 360 individual cells for both cell lines. (unpaired two-tailed *t* test, *p* = 0.7629 for the MDA-MB-231 cells and *p* = 0.1116 for the MCF-7 cells). (**C**) and (**D**) Effects of the PPMPs on the ability of wound healing in the MDA-MB-231 and MCF-7 cell lines. Photos in each group were taken at 0 hours and 12 hours after the PPMP treatment. The closed area (indicated yellow line) was quantified by Image J software. Experiments were repeated independently three times for the MCF-7 cells and five times for the MDA-MB-231 cells (unpaired two-tailed *t* test; *p* = 0.0684 for the MDA-MB-231 cells, *p* = 0.7760 for the MCF-7 cells).

Cancer metastasis is associated with cellular migration and motility. To investigate whether the PPMP exposure affected the migration of human breast cancer cells, PPMPs were administered to hormone-dependent MCF-7 (ER+, PR+, HER2–) and hormone-independent triple-negative MDA-MB-231 (ER–, PR–, HER2–) cells. The cell motility was observed by time-lapse imaging over six hours. The plot shows the path and location of the cell movement by the line from the center (0, 0) (Fig. 3). The rates of 360 cells in each group were quantified and displayed in the graph. As a result, the exposure to PPMPs in the MDA-MB-231 cells did not affect the direction and speed of the cancer cell motility. These results were consistent in both the MDA-MB-231 and MCF-7 cells (Fig. 3A,B).

Further, the wound-healing ability of both breast cancer cell types, MDA-MB-231 and MCF-7, was examined. The cell migration analysis was performed after exposing the cells to 1.6 mg/ml PPMPs for 12 hours. Similarly, the presence of PPMPs did not alter the tendency of wound closure in the MDA-MB-231 and MCF-7 cells (Fig. 3C,D). These results suggest that PPMP exposure does not affect cancer cell motility regardless of the properties of the breast cancer cells.

### PPMPs induced transcriptional changes in RNA-seq analysis

**Figure 4:**
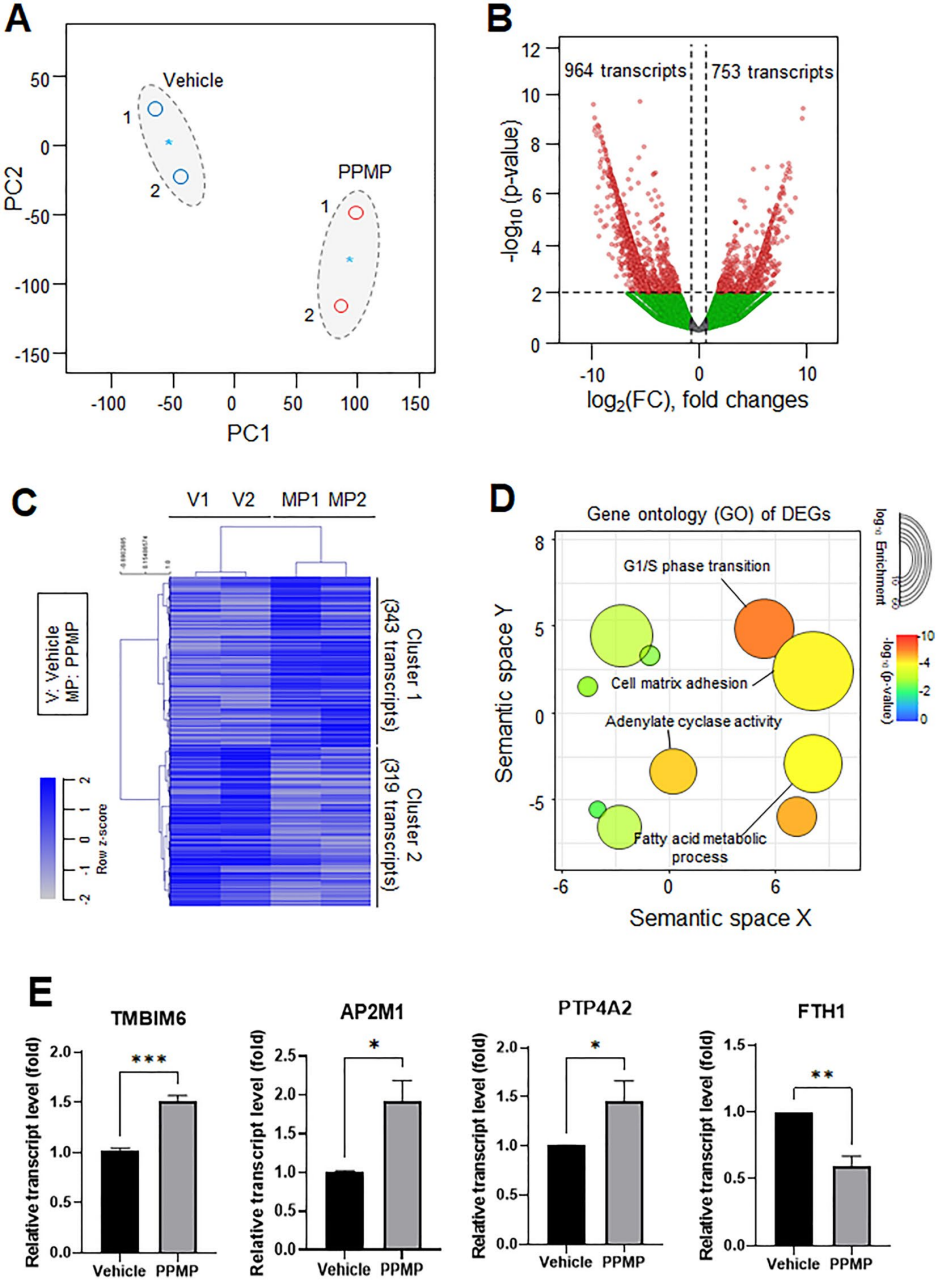
Transcriptomic dynamics in PPMP-treated MDA-MB-231 cells. The common gene expression patterns through two independent experiments were identified by transcriptomic analysis using the RNA-seq platform. (**A**) Principal component analysis (PCA) of the RNA-seq analysis. The values indicate the amount of variation attributed to each principal component. The small circles indicate individual samples, blue asterisks indicate the average between experimental replicates, and larger gray ovals represent each experimental group. (**B**) The volcano plot from the filtered significant DETs from the MDA-MB-231 cells with or without the PPMP incubation. (**C**) The differential gene expression patterns of DETs in the RNA-seq analysis. The hierarchical clustering of genes (rows) and experimental samples (columns) from the RNA-seq data. (**D**) The top enriched GO cellular components and biological processes of PPMP-treated cells, visualized from the significantly upregulated DETs from the RNA-seq platform. The size of the circle represents the enriched genes, and the color represents − log_10_ of the *p*-value. (**E**) Transcript levels of TMBIM6, AP2M1, PTP4A2, and FTH1 were evaluated by qRT-PCR in MDA-MB-231 cells (unpaired two-tailed *t* test, *p < 0.05, **p < 0.01, ***p < 0.001 relative to the vehicle group).

An RNA-seq was performed on the MDA-MB-231 cells with or without PPMP incubation to investigate whether the 1.6mg/ml PPMPs affected gene expression related to breast cancer progression. After the read alignment and quantification, the initial evaluation of the dataset was performed. Using 18,000 of the most expressed transcripts and a principal component analysis (PCA) plot, we assessed the differences among the samples collected from cells with or without PPMPs (Fig. 4A). The first and second principal components accounted for the largest variance among the datasets generated from the RNA-Seq dataset. The application of EdgeR with conservative access to the RNA-seq data gained from the PPMP-treated samples confirmed that there were 1717 differentially expressed transcripts (Fig. 4B). The volcano plots show the statistical significance of the differentially expressed transcripts (DETs) with the respective fold-changes (*p* < 0.01, absolute log2 fold-change [log2 FC] > 1) compared with the control group. The significant upregulation (753 transcripts) or downregulation (964 transcripts) of genes was revealed in the PPMP-treated breast cancer cells (Fig. 4B). Hierarchical clustering of the upregulated (343) or downregulated (319) DETs in the PPMP-treated cells were classified into two categories with stringent filtering conditions (*p* < 0.01, log2 FC > 1.5) (Fig. 4C). The top enriched gene ontology (GO) cellular components and biological processes of the PPMP-exposed cells were visualized from the significant DETs of the upregulated genes as cluster 1 of Fig. 4C. Following the GO enrichment analysis, the cell cycle, cell-matrix adhesion, adenylate cyclase activity, and fatty acid metabolic processes were revealed to be the most significantly enriched pathways in the PPMP-treated cells (Fig. 4D). Among them, G1/S phase transition-related transcript levels were the most highly impacted by the PPMP exposure. Thus, we evaluated the expression of cell cycle-related genes by qRT-PCR. The selected genes were analyzed from cells exposed to PPMPs for 24 hours. The expression of transmembrane BAX inhibitor motif containing 6 (TMBIM6) was about 1.52-fold higher in cells treated with PPMPs than in the vehicle-treated cells (Fig. 4E). The expression of adaptor related protein complex 2 subunit mu 1 (AP2M1) was increased by about 1.92-fold in cells treated with PPMPs. Also, the transcript level of protein tyrosine phosphatase 4A2 (PTP4A2) was increased by about 1.46-fold in the PPMP-treated cells compared with the vehicle-treated cells. However, the expression of the ferritin heavy chain 1 (FTH1) was reduced by about 0.6-fold. These results show that the expression of genes related to the cell cycle was affected by the PPMPs. Interestingly, these changes of gene expression by PPMP were not observed in normal breast epithelial cells, MCF10A. PPMP incubation could only affect breast cancer cells, not normal breast cell line (Supplementary Fig. 1).

**Figure 5:**
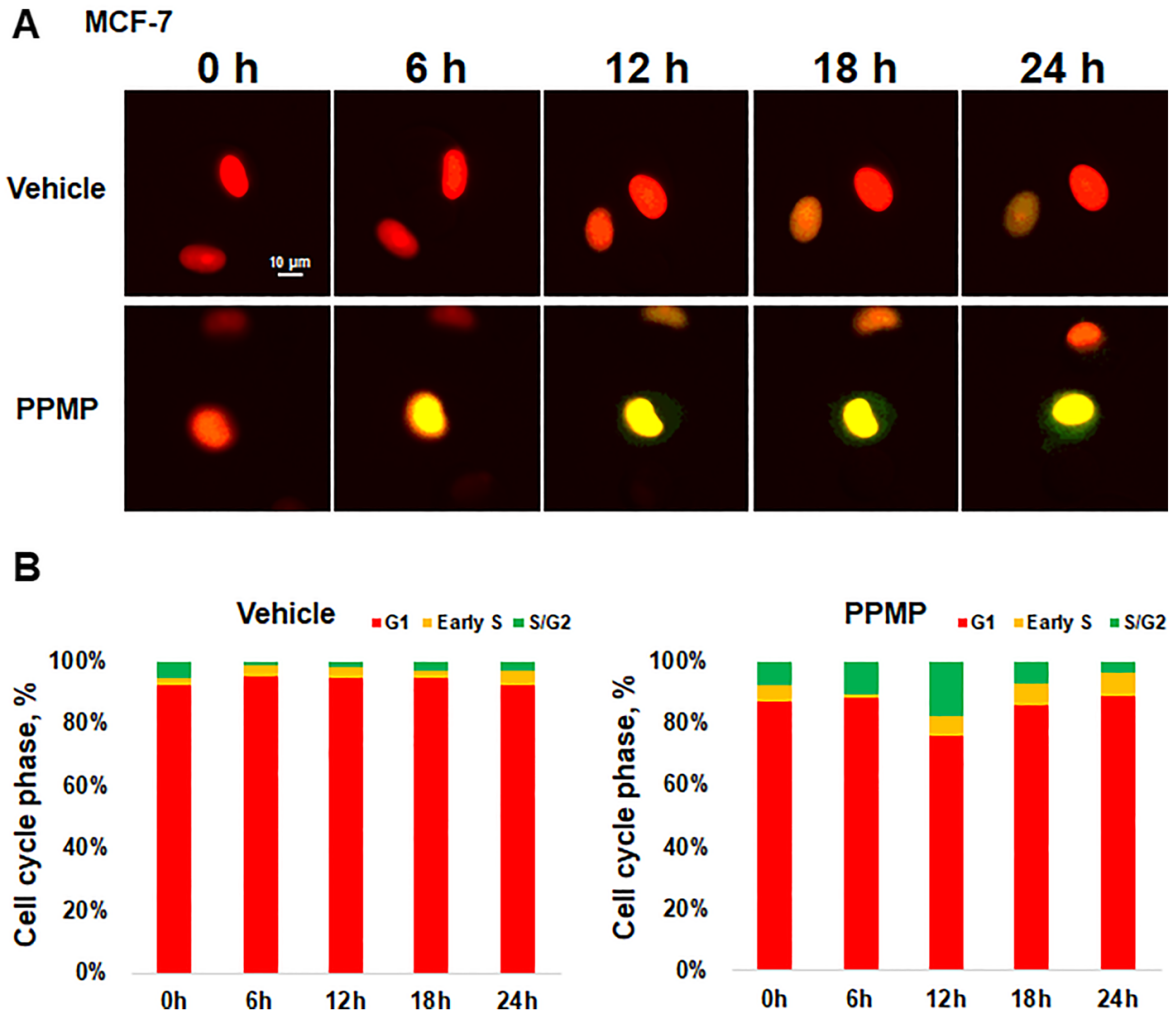
Effect of PPMP on cell cycle in breast cancer cells. (**A**) FUCCI images were captured after the PPMP application at 0, 6, 12 and 24 hours while monitoring the cell cycle of living cells in real-time. The scale bar represents 10 µm. (**B**) Histograms show the cell cycle rate at each time. Cells at each cycle stage were quantitatively evaluated by counting the number of cells of each color. Cell cycle profiles are color coded: G1, red; initial S, yellow; and late S/G2/M, green (n = 52–129 per group total number of cells).

Further, the cell cycle changes in breast cancer cells after exposure to PPMPs were monitored using FUCCI, a cell cycle sensor, for 24 hours. Compared with the vehicle, the color change was accelerated by the PPMPs from red, indicating G1 phase, to yellow, indicating early S phase (Fig. 5A). In Fig. 5B, the cell cycle phases are expressed as percentages at six-hour intervals. After six hours of PPMP treatment, the proportion of cells in the S/G2/M phase was increased, suggesting that PPMPs promote cell cycle changes in breast cancer.

### PPMPs increased the secretion of pro-inflammatory cytokine IL-6 from cancer cells

**Figure 6:**
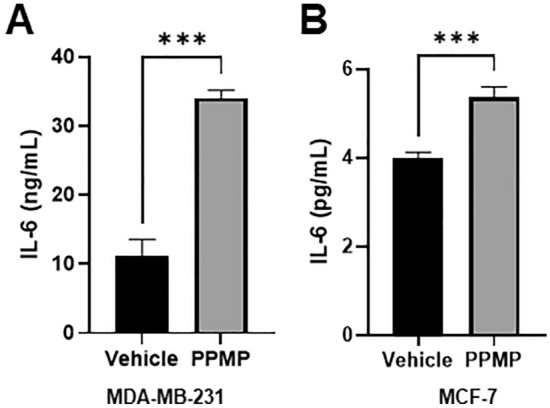
Effect of PPMP on pro-inflammatory cytokines in breast cancer cells. (**A**) and (**B**) IL-6 secretion was evaluated by an ELISA in MDA-MB-231 and MCF-7 breast cancer cells (unpaired two-tailed *t* test, ***p < 0.001, n = 3 for the MDA-MB-231 cells; n = 6 for the MCF-7 cells).

Cytokines are important mediators in the tumor microenvironment and can function as growth factors to increase cell metastasis by promoting extracellular matrix degradation and angiogenesis. In particular, IL-6 is a well-known pro-inflammatory cytokine associated with the tumor microenvironment and has been reported to promote cell cycle progression, proliferation, anti-apoptosis, and metastasis in breast cancer^15–17^. Since the PPMPs promoted the cell cycle of breast cancer cells, we evaluated whether the secretion of IL-6 was increased in cancer cells exposed to 1.6mg/ml PPMPs. After exposure to the PPMPs for 24 hours, the secretion of IL-6 was 34.62 ± 1.41 ng/ml, which was significantly increased compared with the vehicle in MDA-MB-231 cells (*n* = 3, 10.60 ± 3.38 ng/ml). Similarly, the secretion of IL-6 in the MCF-7 cells was also markedly increased in the PPMP group (5.36 ± 0.23 pg/ml) compared with the vehicle group (3.98 ± 0.13 pg/ml) (Fig. 6A,B).

## Discussion

MPs are rapidly emerging as a global challenge that raises concerns about human health and the whole ecosystem. In addition, the increased use of single-use plastics during the COVID-19 pandemic accelerated an already out-of-control global plastic waste problem.

Most of the research on MPs is confined to the effect of the sphere type MPs; however, most of the MPs in the environment are the fragment and fiber type MPs. Therefore, the results of studies on the sphere type MPs are prone to provide biased information on the MP risk assessment to human health. To date, studies on the effect of shape-dependent MPs are very limited. In this study, we focused on the pathophysiological effect of fragmented MPs on cancer cell lines*.* The PPMPs used in this study were a mixture of fragmented PPMPs of various sizes, but the majority had a diameter of 16.4 µm. We demonstrated that the fragmented PPMPs promoted the cell cycle, proliferation, and secretion of the inflammatory cytokine IL-6 in human breast cancer cells, while PPMPs did not induce cytotoxicity nor enhance cellular motility.

Various sized MPs can affect cells differently in vitro or in vivo. MPs are reported to have a wide range of sizes in freshwater and marine environments, varying from 10 µm to 5 mm^18,19^. The tiniest MPs are called nanoplastics and are smaller than 0.1 µm. The accumulation of plastic particles, between the sizes of 5 and 110 µm, in animals, such as rats, dogs, and pigs, was reported as early as 1970^20^. Recently, several studies have reported how plastics might be harmful in vitro and in vivo and that the size of the MPs determines their uptake efficiency through the gastrointestinal, alveolar, and dermal epithelium^21–23^. Although more than 90% of the MPs were reported to be excreted in feces, especially larger particles (> 150 µm), smaller MPs may be absorbed systematically. It has been reported that MPs 0.1–10 µm in size can cross the blood-brain barrier^24^ and the placenta^25^, particles smaller than 150 µm can cross the gastrointestinal epithelium, and MPs smaller than 2.5 µm can enter the systemic circulation system through endocytosis^26^. Thus, the PPMPs in this study were small enough to cross the gastrointestinal tract and travel through the body.

In this study, we applied PPMPs. Polypropylene particles are thought to be harmless polymers according to the guidelines from the Food and Drug Administration (FDA)^27^; however, some studies show that their toxicity is mainly due to heat^28^ and size^29^. In this study, only a few portion of PPMPs among the 1.6 mg/ml of PPMPs were sitting down to the cell surface or bottom of the culture plate (Fig. 2), while most of the PPMPs were floating in the medium. Moreover, our ultrastructural analysis showed that most of the PPMPs could not enter the cells. Interestingly, the PPMPs sunken between the cell membrane or bottom of the plate did not interrupt the cellular migration or motility of cancer cells (Fig. 3). Instead, the PPMPs enhanced IL-6 secretion and the cell cycle of breast cancer cells. Based on the GO of the DEGs from the RNA-seq analysis in our study, G1/S phase transition and cell-matrix adhesion related pathways were activated (Fig. 4D and Supplementary Fig. 2). We also found that the gene expression of TMBIM6, AP2M1, and PTP4A2, which are correlated with cancer progression and the cell cycle^30–32^, was significantly increased by PPMP incubation in MDA-MB-231 human breast cancer cells, suggesting that PPMPs regulate the transcriptional activity of cancer cells.

Interestingly, chemical factors could not be detected in our system, although most of the PPMPs were floating in the culture medium (Fig. 1C). In general, the chemical compounds from MPs, which are used in most plastic processing, are also important factors to be considered. Manufacturers add compounds such as plasticizers, stabilizers, and pigments to plastics and many of these substances are hazardous; for example, they can interfere with the endocrine system^33^. There are also studies showing that benzophenone-A, a plastic stabilizer, stimulates the proliferation of ovarian cancer and breast cancer^34^, and several other chemicals have been shown to have various effects on cell cycle, proliferation, and the reproductive system^35,36^.

Based on the results in Fig. 1C, we concluded that the physical effect of PPMPs on the breast cell line drives cancer progression rather than the chemical effect. PPMPs attached to the cell membrane or inserted between cells may alter the physical contact or stimuli in human breast cancer cells, followed by the enhancement of cancer progressions, such as the cell cycle and IL-6 secretion. However, the chemical effect of PPMPs cannot be excluded. Since the cell culture medium is generally composed of a complement of amino acids, vitamins, inorganic salts, glucose, and serum, which are large-sized biological molecules, there might have been technical limitations in the detection of small-sized chemicals. The exact mechanisms of MPs should be investigated further. Overall, our results demonstrate that PPMPs can promote tumor progression by promoting the inflammatory cytokine IL-6 secretion and cell cycle progression in breast cancer cells. These data support new evidence of the potential risks of MPs.

## Conclusions

In this study, we observed that PPMPs with irregular shapes affected human breast cancer cells; notably, exposure to PPMPs increased cell cycle-related gene expression and IL-6 secretion from MDA-MB-231 and MCF7 cells. Moreover, 1.6 mg/ml of PPMPs used in this study might be a high concentration for cancer cells, but this concentration neither induced cell death nor altered the migration ability of breast cancer cells. Additionally, the RNA-seq analysis revealed that PPMPs promoted metastatic features in cancer cells instead.

Although many researchers have reported the detrimental effects of MPs on our health, a comprehensive analysis of the chemical and physical effects of MPs has yet to be conducted. Here, we demonstrated for the first time the effect MPs have on human breast cancer. During and after the COVID-19 pandemic, the indiscriminate use of plastics may exacerbate the harmful effects of MPs on our health. Therefore, this study is a starting point for the discussion between the chronic exposure to MPs and cancer progression.

## Supplementary Information

Supplementary Figures.

## Data Availability

All data analyzed during this study are included in this published article and its supplementary information file. The datasets used in Fig. 4 and supplementary information are downloaded from Gene Expression Omnibus (GEO) database under accession number GSE219207.

## Abbreviations

MP: Microplastic
SEM: Scanning electron microscopy
PBS: Phosphate buffered saline
HMDS: Hexamethyldisilazane
HPLC/DAD: High-performance liquid chromatography/diode array detector
qRT-PCR: Quantitative real-time polymerase chain reaction
ELISA: Enzyme-linked immunoassay
ER: Estrogen receptor
PR: Progesterone receptor
HER2: Human epidermal growth factor receptor 2
