# Supplementary material for: Polypropylene microplastics promote metastatic features in human breast cancer
Source: Sci Rep. 2023 Apr 17;13:6252. doi: 10.1038/s41598-023-33393-8 (PMC10108816; doi:10.1038/s41598-023-33393-8)
Supplement: Supplementary file 1 — Supplementary Figures. [file 41598_2023_33393_MOESM1_ESM.docx]

**Supplementary Information**


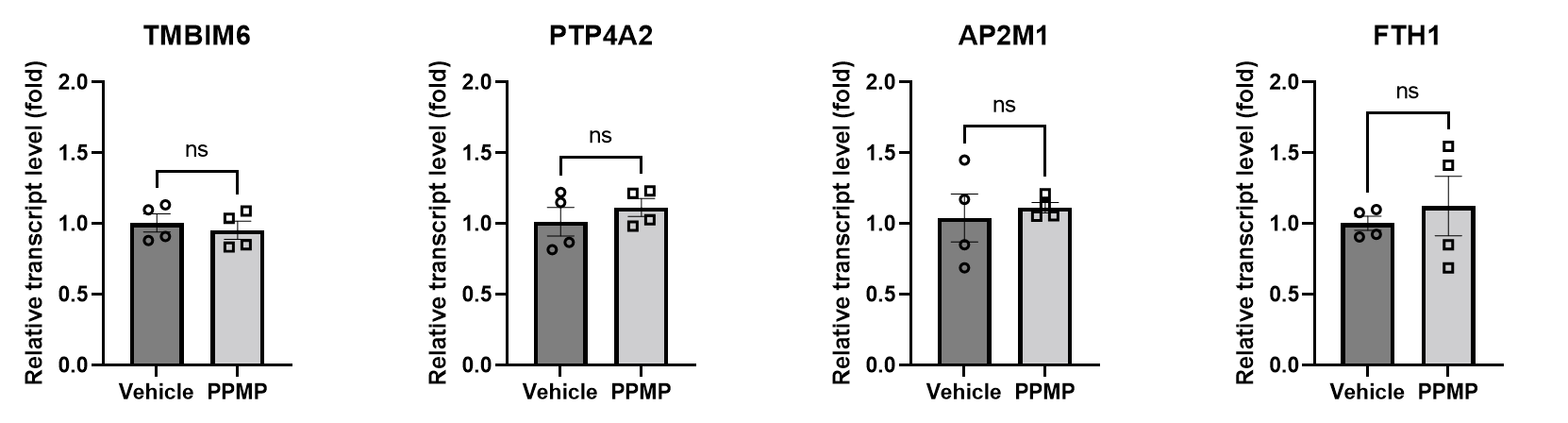


**Supplementary Figure 1. Cell cycle related gene expression by PPMP incubation with MCF 10A cells.**

Human normal breast epithelial cell line, MCF 10A cells were incubated with PPMPs for 24 h and then, transcript level of TMBIM6, AP2M1, PTP4A2, and FTH1 were evaluated by qRT-PCR in MCF 10A cells (unpaired t-test, n.s., not significant).


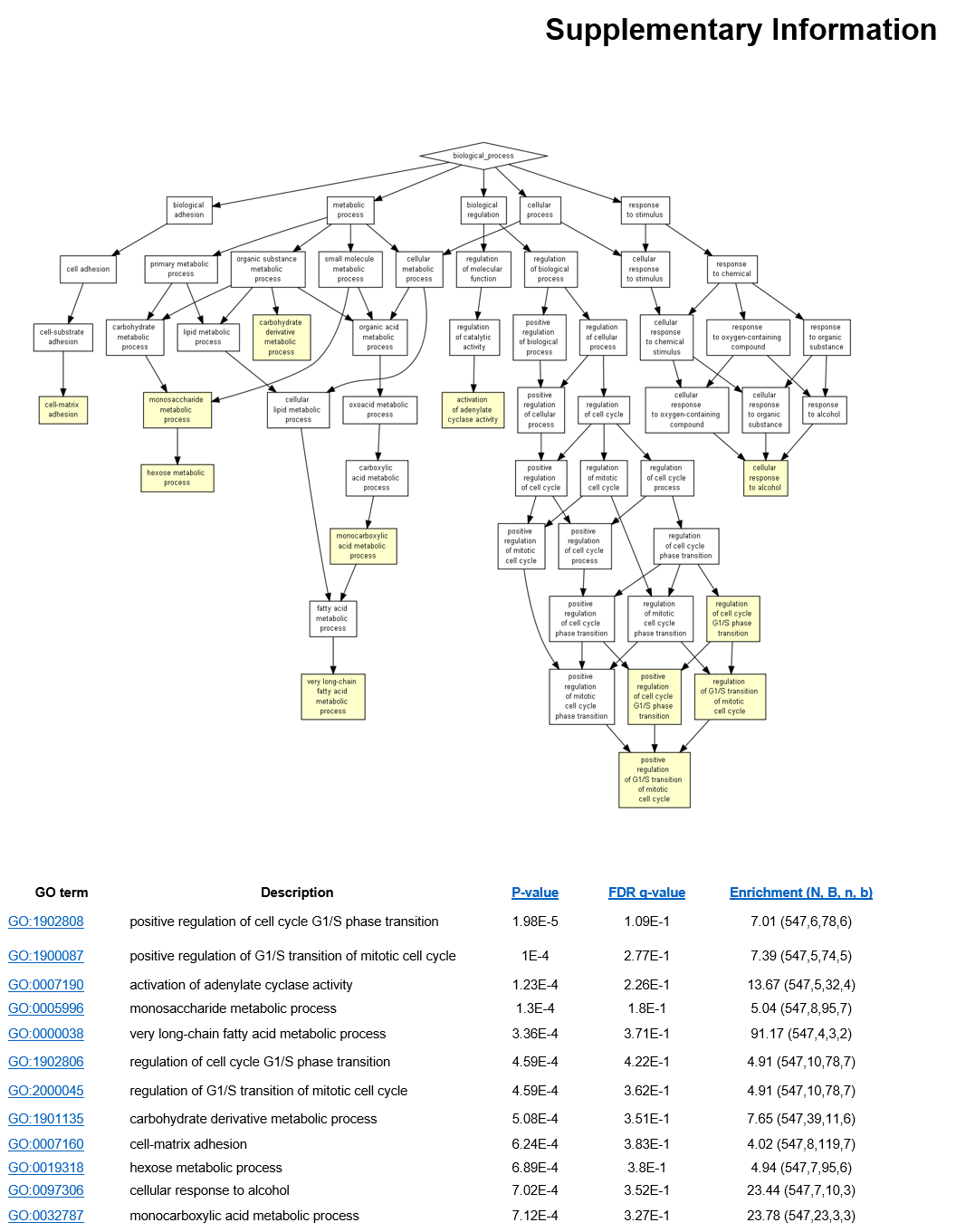


**Supplementary Figure 2. Activated pathways by PPMP incubation with MDA-MB-231 cells.**

Based on the GO of the DEGs from the RNA-seq analysis, G1/S phase transition, mitotic cell cycle, and cell-matrix adhesion related pathways were activated by PPMP incubation with breast cancer cells.
